# Axonal BACE1 dynamics and targeting in hippocampal neurons: a role for Rab11 GTPase

**DOI:** 10.1186/1750-1326-9-1

**Published:** 2014-01-04

**Authors:** Virginie Buggia-Prévot, Celia G Fernandez, Sean Riordan, Kulandaivelu S Vetrivel, Jelita Roseman, Jack Waters, Vytautas P Bindokas, Robert Vassar, Gopal Thinakaran

**Affiliations:** 1 Department of Neurobiology, The University of Chicago, Chicago, IL 60637, USA; 2 Department of Neurology, The University of Chicago, Chicago, IL 60637, USA; 3 Department of Pathology, The University of Chicago, Chicago, IL 60637, USA; 4 Department Neurobiology, Pharmacology and Physiology, The University of Chicago, Chicago, IL 60637, USA; 5 Committee on Neurobiology, The University of Chicago, Chicago, IL 60637, USA; 6 Department of Cell and Molecular Biology, Northwestern University, Chicago, IL 60611, USA; 7 Department of Physiology, Feinberg School of Medicine, Northwestern University, Chicago, IL 60611, USA

**Keywords:** BACE1, Rab11, Transcytosis, Axonal sorting, Axonal transport, Recycling endosome

## Abstract

**Background:**

BACE1 is one of the two enzymes that cleave amyloid precursor protein to generate Alzheimer's disease (AD) beta amyloid peptides. It is widely believed that BACE1 initiates APP processing in endosomes, and in the brain this cleavage is known to occur during axonal transport of APP. In addition, BACE1 accumulates in dystrophic neurites surrounding brain senile plaques in individuals with AD, suggesting that abnormal accumulation of BACE1 at presynaptic terminals contributes to pathogenesis in AD. However, only limited information is available on BACE1 axonal transport and targeting.

**Results:**

By visualizing BACE1-YFP dynamics using live imaging, we demonstrate that BACE1 undergoes bi-directional transport in dynamic tubulo-vesicular carriers along axons in cultured hippocampal neurons and in acute hippocampal slices of transgenic mice. In addition, a subset of BACE1 is present in larger stationary structures, which are active presynaptic sites. In cultured neurons, BACE1-YFP is preferentially targeted to axons over time, consistent with predominant *in vivo* localization of BACE1 in presynaptic terminals. Confocal analysis and dual-color live imaging revealed a localization and dynamic transport of BACE1 along dendrites and axons in Rab11-positive recycling endosomes. Impairment of Rab11 function leads to a diminution of total and endocytosed BACE1 in axons, concomitant with an increase in the soma. Together, these results suggest that BACE1 is sorted to axons in endosomes in a Rab11-dependent manner.

**Conclusion:**

Our results reveal novel information on dynamic BACE1 transport in neurons, and demonstrate that Rab11-GTPase function is critical for axonal sorting of BACE1. Thus, we suggest that BACE1 transcytosis in endosomes contributes to presynaptic BACE1 localization.

## Background

A transmembrane aspartyl protease, termed β-site APP cleaving enzyme 1 (BACE1), initiates Alzheimer's disease (AD) β-amyloid (Aβ) production by sequential proteolysis of amyloid precursor protein (APP) [1-5]. The release of Aβ from its precursor involves initial processing by BACE1 to release the APP ectodomain, followed by intramembrane proteolysis by γ-secretase [6]. Aβ is the major component of cerebral amyloid plaques in brains of aged individuals and those with AD. Several lines of evidence suggest that Aβ plays a central role in AD pathogenesis. For example, familial AD-linked mutations near the amino terminus of the Aβ region in APP, found in two Swedish families, cause AD by significantly increasing Aβ production due to enhanced BACE1 cleavage of APP [7-9]. Moreover, a single amino acid substitution adjacent to the BACE1 cleavage site of APP, which significantly reduces BACE1 cleavage and Aβ peptide generation in cultured cells, has been recently found to protect against disease onset as well as cognitive decline in the elderly without AD [10].

APP and BACE1 are type I transmembrane proteins that undergo secretory and endocytic trafficking. However, in cultured cell lines and primary neurons, only a subset of full-length APP is processed to generate Aβ. This implies either BACE1 cleavage of APP is rather inefficient or that BACE1 has limited access to APP due to their distinct intracellular itineraries and/or spatially restricted localization in intracellular organelles. Over the years, non-neuronal cells were used as experimental systems to characterize the cellular organelles and sorting pathways involved in amyloidogenic processing of APP. Most studies recognize the importance of endocytic trafficking of APP for Aβ production [reviewed in [6,11,12]. This notion is in agreement with the predominant steady-state localization of BACE1 in endocytic organelles, and its trafficking and recycling between the plasma membrane and endosomes [1,13,14]. Moreover, BACE1 activity has a low pH optimum *in vitro*[1], supporting a model where APP cleavage by BACE1 occurs in acidic intracellular organelles such as the endosomes. Indeed, siRNA knockdown of Golgi-associated, γ-adaptin homologous, ADP-ribosylation factor-interacting proteins enhance BACE1 localization in early endosomes and a concomitant increase in Aβ secretion [15-17]. Finally, membrane targeting of a BACE1 transition-state inhibitor to endosomal organelles by linking it to a sterol moiety efficiently reduced BACE1 activity as compared with the free inhibitor [18].

In the mouse brain, ~70% of Aβ released into the interstitial fluid requires ongoing endocytosis, and synaptic activity regulates the vast majority of this endocytosis-dependent Aβ production [19,20]. The underlying mechanism likely involves activity-dependent regulation of endocytic trafficking of APP as well as its secretases [21]. Neuronal endosomes are not only found in the soma but are distributed throughout the dendrites and axons where they undergo bidirectional transport, adding to the complexity of the trafficking mechanisms [22]. Indeed, APP undergoes BACE1-mediated cleavage during anterograde axonal transport, and Aβ can be generated and released at or near presynaptic sites *in vivo*[19,23-27]. BACE1 has been reported to localize in dendrites and axons in cultured neurons and in the brain [21,28-32]. Axonal BACE1 localization is significant because abnormal accumulation of BACE1 in axon terminals has been documented in the brains of individuals afflicted with AD. This later finding raises the possibility that local elevation in BACE1 processing could contribute to amyloid burden in AD [30,33]. However, the molecular mechanisms responsible for axonal sorting of BACE1 have not been fully explored.

Here, we used live-cell imaging to characterize dynamic BACE1 transport in hippocampal neurons *in vitro* and in brain slices *in situ*. We report BACE1 colocalization and dynamic transport in recycling endosomes within the dendrites and axons of cultured hippocampal neurons. Interestingly, our results show that efficient axonal sorting of BACE1 requires Rab11 activity. Together, these findings provide the first demonstration of dynamic BACE1 transport in hippocampal neurons *in situ* and the involvement of neuronal recycling endosomes in axonal sorting of BACE1.

## Results

### Axonal transport and presynaptic localization of BACE1 in hippocampal mossy fibers

**Figure 1:**
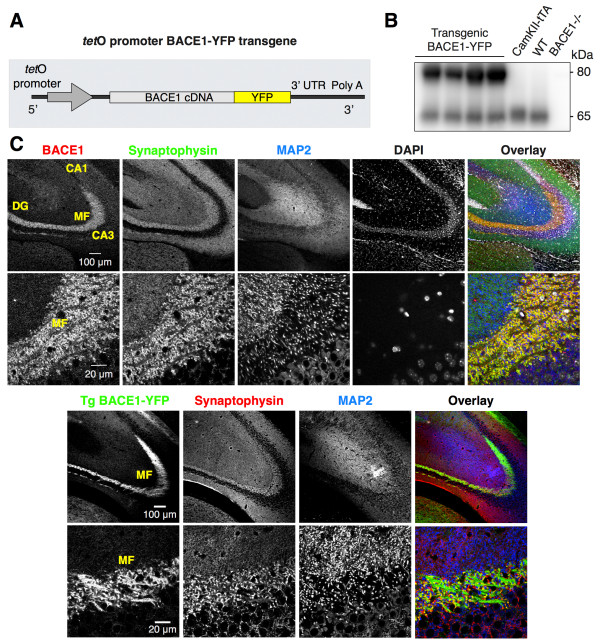
**Axonal and presynaptic localization of BACE1-YFP in hippocampal mossy fibers of transgenic mice. (A)** Schematic structure of *tet*O BACE1-YFP transgene. **(B)** BACE1 expression in mouse forebrain lysates from bigenic BACE1-YFP (Tg BACE1-YFP), CamKIIα-tTA, non-transgenic (WT), and BACE1−/− mice was analyzed by immunoblotting with mAb 3D5. **(C)** Localization of endogenous BACE1 and BACE1-YFP in presynaptic terminals of the mouse brain hippocampal mossy fibers (MF). Top, non-transgenic mouse brain stained with anti-BACE1 mAb 3D5. 3 month-old non-transgenic (top panel) or Tg BACE1-YFP mouse brains (bottom) were stained with antibodies against synaptophysin, and MAP2 and were analyzed by confocal microscopy. Neuronal cell bodies in dentate gyrus (DG) were only weakly positive for BACE1-YFP.

**Figure 2:**
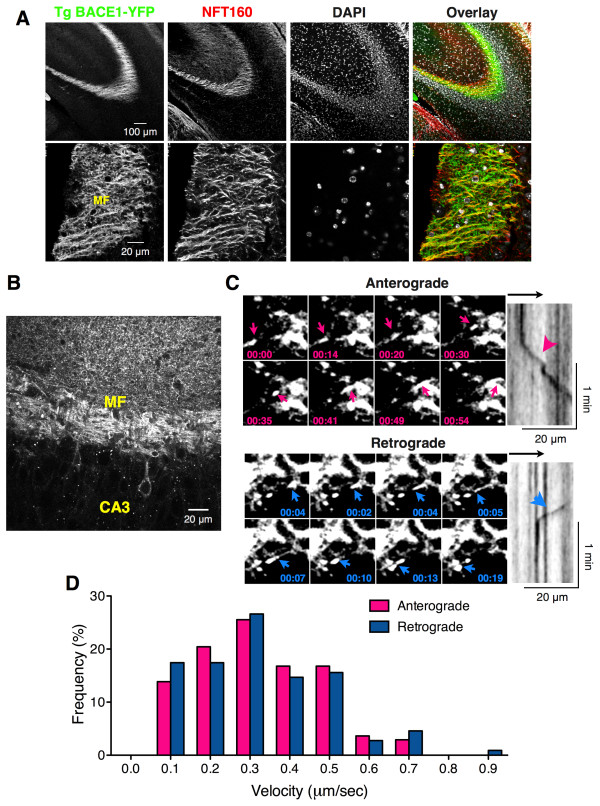
**Dynamic transport of BACE1-YFP in hippocampal mossy fibers of Tg BACE1-YFP mice. (A)** Tg BACE1-YFP brain stained with anti-neurofilament NFT160 mAb. Note the prominent localization of BACE1-YFP in axons of hippocampal mossy fibers (MF). Nuclei of neuronal cell bodies were labeled using DAPI staining. **(B)** Representative 2-photon confocal image of YFP fluorescence in Tg BACE1-YFP hippocampal brain slice acquired near the region used for time-lapse imaging depicted in **(B)**. MF, Mossy fibers. Note BACE1-YFP fluorescence in puncta of dendrites and in the soma of a few CA3 neurons. **(C)** Time-lapse images of hippocampal slices from Tg BACE1-YFP were acquired at 30°C on a multiphoton confocal microscope at the rate of 0.8 frames/sec for 4 min. Montage of representative images from Additional file 1, and corresponding kymographs are shown. Arrowheads indicate the movement of BACE1-positive vesicular/tubular carriers away from the DG (anterograde) or toward the DG (retrograde). **(D)** Maximum velocities of BACE1-YFP carriers (n = 136 anterograde; n = 113 retrograde; 8 brain slices from 3 BACE1-YFP bigenic mice imaged) were quantified from kymographs and plotted as a histogram.

In the mouse brain, BACE1 exhibits prominent localization in the *stratum lucidum* of the hippocampus, composed of axons and presynaptic terminals of mossy fibers from granule cells in the dentate gyrus, and is only weakly detected in dendrites [28,30,33]. Although endogenous BACE1 is not detected along the axons (Figure 1C), localization of BACE1 in large terminals of hippocampal mossy fibers suggests that BACE1 traffics to mossy fiber terminals by axonal transport. To visualize dynamic BACE1 axonal transport in mossy fibers, we generated bigenic mice in which yellow fluorescent protein-tagged BACE1 (BACE1-YFP) is inducibly expressed under the control of the forebrain-specific CaMKIIα promoter (Figure 1A and 1B). In control experiments, we confirmed that BACE1-YFP chimeric protein is able to process APP with efficiencies similar to wild-type BACE1 [34]. Similar to endogenous BACE1 localization, BACE1-YFP fluorescence in brains of bigenic mice was prominent within the hippocampal mossy fibers. A subset of BACE1-YFP fluorescence appeared in punctate structures, which colocalized with the presynaptic marker synaptophysin (Figure 1C). In addition, BACE1-YFP fluorescence was visible along the axons, and showed partial colocalization with neurofilament (Figure 2A). Thus, similar to endogenous BACE1, transgene-derived BACE-YFP is able to reach the presynaptic terminals. Since BACE1-YFP is overexpressed a few fold over endogenous levels, there is likely more BACE1-YFP in transit along hippocampal mossy fibers, which could be seen as axonal localization.

In order to visualize dynamic BACE1 axonal transport in the mossy fiber axons, acute hippocampal slices of bigenic mice were maintained at 30°C and live imaging was performed. Multi-photon confocal imaging of the *stratum lucidum* revealed BACE1-YFP fluorescence along the mossy fibers within stationary structures and in tubulo-vesicular carriers, which displayed alternating dynamic movement and pausing behavior (Figures 2B and 2C, Additional file 1). Kymographs were generated to analyze BACE1-YFP transport dynamics. Transport of BACE1-YFP along the mossy fiber axons could be observed in both the anterograde and retrograde directions (away from and towards the dentate gyrus hilus, respectively) with velocities ranging from 0.1 – 0.7 μm/sec (Figure 2D). The mean velocities for anterograde and retrograde movement of BACE1-YFP-containing carriers were very similar (anterograde 0.325 ± 0.013 μm/sec, n = 137; retrograde 0.330 ± 0.016 μm/sec, n = 109). Importantly, these results represent the first demonstration of dynamic axonal transport of BACE1 in mature CNS neurons *in situ*.

### Axonal localization and transport of BACE1 in cultured hippocampal neurons

**Figure 3:**
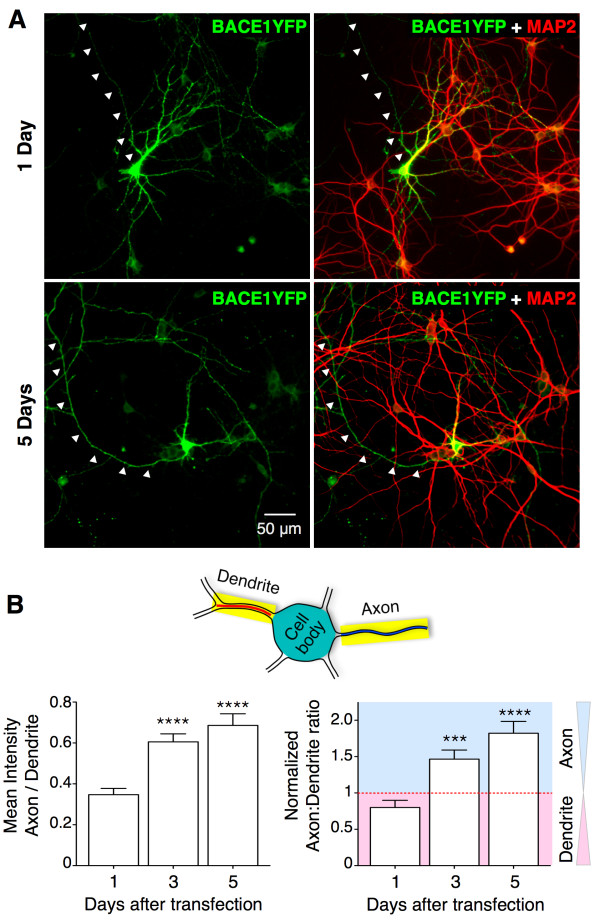
**Preferential axonal targeting of BACE1-YFP over time after transfection in cultured hippocampal neurons. (A)** Cultured hippocampal neurons were co-transfected with plasmids expressing BACE1-YFP and Cerulean on DIV5 (when they have already developed dendrites and long axons) and polarized distribution of BACE1-YFP was analyzed after 1, 3, and 5 days of transgene expression. Representative images of BACE1-YFP distribution neurons 1 or 5 days after transfection are shown. MAP2 staining was used to identify dendrites and, by exclusion, axons (*arrowheads*). **(B)** Mean axon-to-dendrite fluorescence intensity ratios were calculated for BACE1-YFP and Cerulean along major axon and dendrite segments in co-transfected neurons analyzed after 1, 3, and 5 days of transgene expression (n = 23-28 neurons each imaged from four coverslips per time point, two independent cultures).

The prominent presynaptic terminal localization of endogenous as well as transgene-derived BACE1 in hippocampal mossy fiber terminal fields suggested the possibility that BACE1 might undergo polarized sorting. However, BACE1 has been previously reported to localize in neuronal soma, dendrites, and axons of neurons in human brain [4,34], and in cultured neurons [21,28-31,35]. In our experiments, BACE1-YFP localization could be readily detected both in dendrites and axons of cultured DIV5 hippocampal neurons following overnight expression (Figure 3A). We reasoned that subtle differences in the biosynthetic level of BACE1 and the efficiency of the transport machinery involved in BACE1 transport could account for these apparent discrepancies. If this were the case, high-level transient protein overexpression in the first one or two days following transfection could be in excess over the trafficking modulators, and might not be representative of the steady-state BACE1 localization in cultured hippocampal neurons. Therefore, we decided to observe BACE1 distribution several days after transfection to assess whether or not there is preferential axonal localization of BACE1 in cultured hippocampal neurons. To this end, we co-transfected DIV5 neurons with BACE1-YFP and Cerulean and observed them after 1, 3 and 5 days of expression (Figure 3A). We used MAP2 staining to distinguish dendrites and axons. In each transfected neuron, we measured the average fluorescence intensities of BACE1-YFP and Cerulean, along 1 pixel-wide line segments traced on 2–3 representative sections of dendrites and axons, and calculated the raw axon:dendrite ratios. Then we divided the raw axon:dendrite ratio of BACE1 by the raw axon:dendrite ratio of Cerulean (which represents non-polarized localization of a cytosolic protein) [36]. This normalization permits one to easily interpret the data because a normalized ratio of >1 indicates preferential axonal targeting, <1 indicates preferential dendritic localization, and a ratio of 1 indicates no preference between localization in the axon or dendrite. Interestingly, we found that the axon:dendrite ratio of BACE1-YFP significantly increased in the days following transfection while in the same neurons the axon:dendrite ratio of Cerulean is not significantly different (Figure 3B). These results indicate a preference for axonal BACE1-YFP targeting over time in cultured hippocampal neurons, consistent with the *in vivo* BACE localization in mossy fiber terminals.

**Figure 4:**
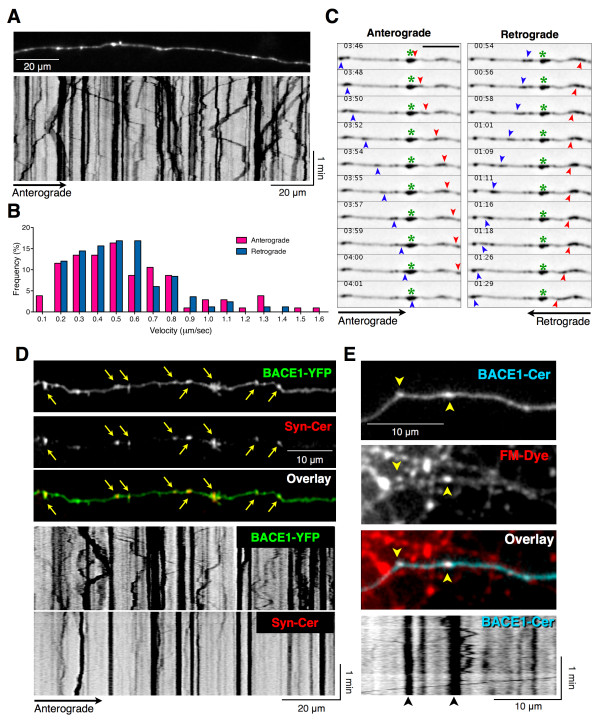
**Characterization of dynamic BACE1 axonal transport and localization in functional synapses in cultured hippocampal neurons. (A-C)** Axonal transport of BACE1-YFP. **(A)** Time-lapse images of transfected neurons (DIV12-13) were acquired at the rate of 1 frame/sec for 3–5 min. A representative frame and the kymograph of the image series corresponding to Additional file 2 are shown. **(B)** Maximum velocities of vesicles moving away (anterograde, n = 106) or toward (retrograde, n = 83) the cell body were quantified from kymographs of multiple axons imaged from five independent cultures and plotted. **(C)** Representative image montages of time-lapse sequences from a segment of the axon shown in Additional file 2 depict dynamic transport of BACE1-containing vesicles and tubulo-vesicular structures. Vesicles containing BACE1-YFP are transported in the anterograde (left panels) or the retrograde (right panels); several BACE1-positive structures also remain stationary (indicated by a green *asterisks*). Note the anterograde BACE1-containing vesicles emerging from the stationary larger vesicular structure. **(D)** Presynaptic localization of BACE1 in cultured hippocampal neurons. Live-cell images from a transfected neuron (DIV14) show colocalization of BACE1-YFP with synaptophysin-Cerulean. Sequential time-lapse images were acquired on the same axon (Additional file 3), and the dynamics of BACE1 and synaptophysin were analyzed by generating kymographs. Note that the large, stationary, BACE1-containing pleomorphic structures are positive for synaptophysin. **(E)** Localization of BACE1-Cerulean (BACE1-Cer) in functional synapses of DIV13 neurons labeled with FM_1-43_ (*arrowheads*). A kymograph of BACE1-Cer dynamics in the same axonal segment is shown in the bottom panel. Note that BACE1-Cer remains stationary (*arrowheads*) in structures that overlap with FM_1-43_ labeled synapses.

To further analyze neuronal BACE1 localization and trafficking, we transfected cultured primary mouse hippocampal neurons with BACE1-YFP on DIV11 and analyzed them after 24–48 h. By performing live-cell imaging of neurons maintained at 37°C, we found that axonal BACE1-YFP resided within stationary structures as well as in tubulo-vesicular carriers, which underwent bidirectional transport (Figure 4A and 4C, Additional file 2). The axonal transport speeds of individual BACE1-YFP carriers varied greatly, but the mean anterograde and retrograde velocities were very similar (anterograde 0.561 ± 0.032 μm/sec, n = 104; retrograde 0.529 ± 0.028 μm/sec, n = 83) (Figure 4B). The transport dynamics of BACE1-YFP are in general agreement with previously described fast axonal transport of syntaxin 13, which ranged from 0.2 – 0.53 μm/sec [37]. Similar to our observations in hippocampal slices, we observed saltatory movement of BACE1-YFP carriers characterized by bursts of rapid movement alternating with periods of brief pause. In addition, several transport vesicles changed their direction of movement, sometimes more than once, after short pauses (Figure 4A and 4C, Additional file 2). Together, the data from acute hippocampal slices and cultured hippocampal neurons indicate that a significant fraction of BACE1-YFP undergoes dynamic axonal transport.

In both acute hippocampal slices and cultured neurons, a fraction of BACE1-YFP fluorescence remained stationary for prolonged periods, and dynamic anterograde and retrograde carriers often paused at these sites along the axons (Figures 2C and 4A, Additional files 1 and 2). Because a significant amount of endogenous BACE1 exhibits presynaptic localization in hippocampal mossy fibers (Figure 1C), we sought to determine whether the stationary BACE1-YFP fluorescence represented presynaptic BACE1 localization. Live-cell imaging and kymograph analysis of transfected neurons showed that most, if not all, “stationary” BACE1-YFP fluorescence corresponded to presynaptic sites marked by the accumulation of co-transfected synaptophysin-cerulean (Figure 4D, Additional file 3). To confirm BACE1 localization in active presynaptic sites, we labeled live DIV14 neurons with the fluorescent styryl pyridinium dye FM_1-43_, which is widely used to visualize synaptic vesicle recycling [38]. FM_1-43_ uptake studies in live neurons transfected with BACE1-Cerulean confirmed that stationary BACE1 fluorescence co-localized with FM_1-43_ fluorescence, indicating that a subset of axonal BACE1 is present in functional synapses (Figure 4E). Together, these results show that BACE1 undergoes dynamic axonal transport in hippocampal slices and in cultured hippocampal neurons, and both endogenous and transgenic BACE1 localize to presynaptic terminals.

### BACE1-YFP is dynamically co-transported in Rab11-positive recycling endosomes

**Figure 5:**
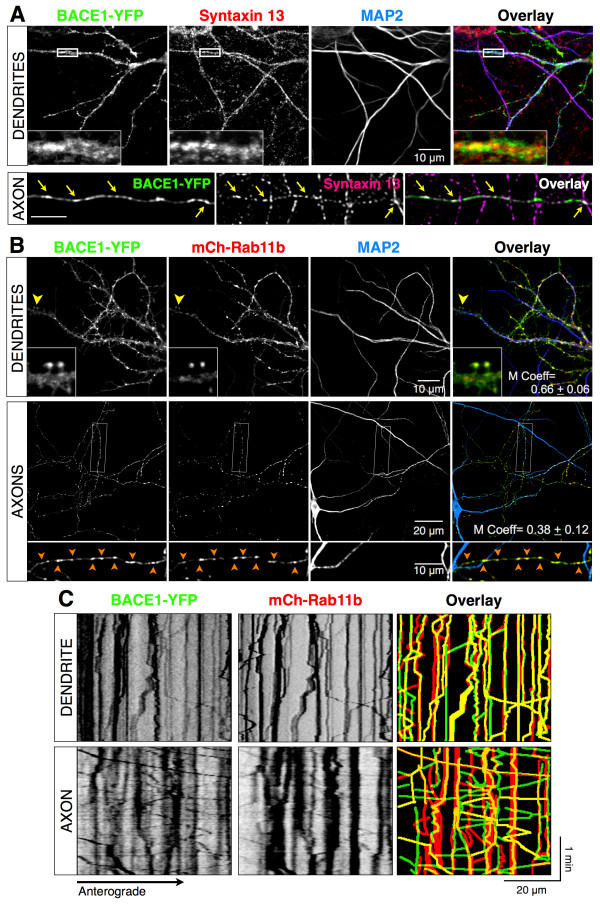
**Localization and dynamic transport of BACE1 in recycling endosomes. (A)** Colocalization of BACE1 with endogenous syntaxin 13 in dendrites and axons of cultured hippocampal neurons (DIV12). Insets show higher magnification of the boxed area. Images of the dendrites were acquired by confocal microscopy and those of the axon were generated by deconvolution of widefield *z*-stacks. Dendrites and axons were identified by the presence and absence of MAP2 staining, respectively. **(B)** Analysis of BACE1 colocalization with Rab11b in dendrites and axons. DIV12 neurons co-transfected with BACE1-YFP and mCherry-Rab11b were immunostained for MAP2. Insets show higher magnification of the area marked by a yellow arrowhead on a dendrite or the boxed area in an axon. *Arrowheads* indicate vesicles positive for BACE1 and Rab11b along the axon. Manders’ coefficient of colocalization (M Coeff) is indicated (n = 7 neurons). **(C)** Visualization of BACE1-YFP and mCherry-Rab11b dynamic co-transport by dual-color live cell imaging. Time-lapse images were acquired using the Dual-View two-channel simultaneous-imaging system at the rate of 1 frame/sec for 3 min (Additional file 4). Kymograph analysis reveals bidirectional transport of BACE1 in Rab11b-positive endosomes (yellow tracks in the overlay panels) in both dendrites and axons.

As mentioned above, the dynamics of BACE1 axonal transport is similar to what has been reported for syntaxin 13, a protein that localizes to recycling endosomes. Indeed, in transfected neurons BACE1-YFP co-localized extensively with endogenous syntaxin 13 in dendrites and in axons (Figure 5A) confirming BACE1 localization in recycling endosomes. To confirm this finding, we assessed co-localization of BACE1-YFP with Rab11, a GTPase that regulates slow recycling of many endocytic cargos [39]. In neurons, Rab11b (the neuron-specific Rab11 isoform) plays key roles in trafficking of proteins such as AMPA receptors, Trk receptors and N-Cadherin in recycling endosomes [40-42]. In co-transfected neurons, BACE1-YFP extensively colocalized with mCherry-Rab11b in dendritic spines, at the bases of spines, and in dendritic shafts [Manders’ coefficient 0.66 ± 0.06], as well as along axons [0.38 ± 0.12] (Figure 5B). To test if BACE1 is dynamically transported in Rab11b-positive vesicles in dendrites and axons, we performed dual-color time-lapse imaging and generated kymographs. This analysis revealed both a dynamic and stationary pool of Rab11b-positive structures. Notably, carriers positive for both BACE1 and Rab11b underwent dynamic bidirectional co-transport in dendrites and axons (Figure 5C, Additional file 4). Thus, in hippocampal neurons, BACE1 localizes to, and is dynamically transported in, Rab11b-positive recycling endosomes along dendrites and axons.

### Axonal polarized sorting of BACE1 requires Rab11 GTPase activity

**Figure 6:**
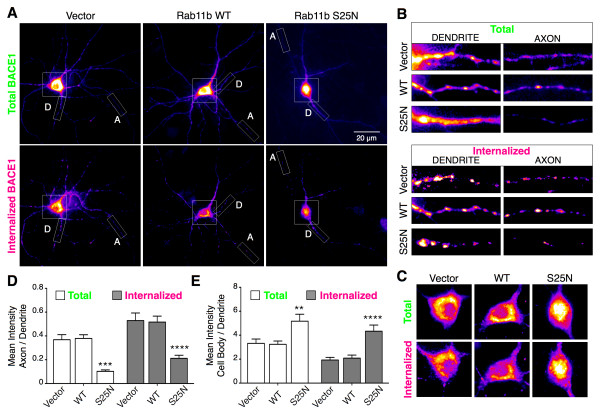
**Rab11b GTPase activity regulates axonal targeting of BACE1. (A)** DIV12 neurons co-transfected with BBS-BACE1-YFP and mCherry-Rab11b WT or Rab11b_S25N_ dominant-negative mutant were labeled with AF647-BTX uptake followed by an acid wash to selectively visualize internalized BACE1. Dendrites and axons were identified by MAP2 immunostaining of fixed neurons. The pseudocolor images depict total (YFP) and internalized BACE1 (BTX) fluorescence intensities in the same neuron. **(B)** Higher magnification of dendrite and axon segments (rectangle boxes) of neurons shown in **A**. **(C)** Higher magnification of the cell bodies (square boxes) of neurons shown in **A**. **(D)** Mean axon-to-dendrite fluorescence intensity ratios for total (YFP) and internalized BACE1 (BTX) were quantified from individual neurons cotransfected with BACE1 and empty vector, Rab11b WT or Rab11_S25N_ (n = 14-15 neurons each). **(E)** Mean cell body-to-dendrite fluorescence intensity ratios for total and internalized BACE1 were quantified. Note that expression of Rab11_S25N_ resulted in a significant decrease of axonal BACE1 localization and an increase of BACE1 levels in the cell body.

Rab11-positive endosomes meditate transcytosis of internalized Trk receptors in sympathetic neurons [41]. In order to examine whether Rab11 plays a role in facilitating polarized sorting of BACE1-YFP in axons of hippocampal neurons, we overexpressed wild-type (WT) Rab11b or a GDP-bound dominant-negative (DN) Rab11b mutant (Rab11_S25N_) together with a BACE1-YFP modified at the N-terminus with the 13-amino acid α-Bungaratoxin Binding Site (BBS) tag, termed BBS-BACE1-YFP. BBS-tagged receptors have been shown to efficiently bind fluorescently labeled α-bungarotoxin (BTX) in live neurons [43]. Recently, we used this strategy to selectively label the internalized pool of BACE1 in dendrites and axons of hippocampal neurons [34]. In neurons transfected with Rab11b, we employed the BTX labeling method to label internalized BACE1-YFP by incubating live neurons at 37°C in presence of Alexa-Fluor 647 labeled α-bungarotoxin (AF647-BTX). After saturable labeling of internalized BACE1 by 3–4 h of continuous AF647-BTX uptake, we removed the remaining surface-bound AF647-BTX using a brief acid wash in a low pH buffer and then quantified the axon:dendrite ratios. BTX-labeling of internalized BACE1 was observed in neurons transfected with empty vector, wt Rab11b, and Rab11b_S25N_ (Figure 6A). However, axon:dendrite ratio quantifications revealed a significant decrease in the levels of total (YFP fluorescence) as well as internalized BACE1 (BTX fluorescence) in axons of neurons expressing Rab11b_S25N_ (Figure 6A, 6B and 6D). We next measured the ratio of the mean fluorescence intensity of the cell body and dendrites and found an increase of BACE1 cell body:dendrite ratio in neurons expressing Rab11b_S25N_ (Figures 6E and 6F).

**Figure 7:**
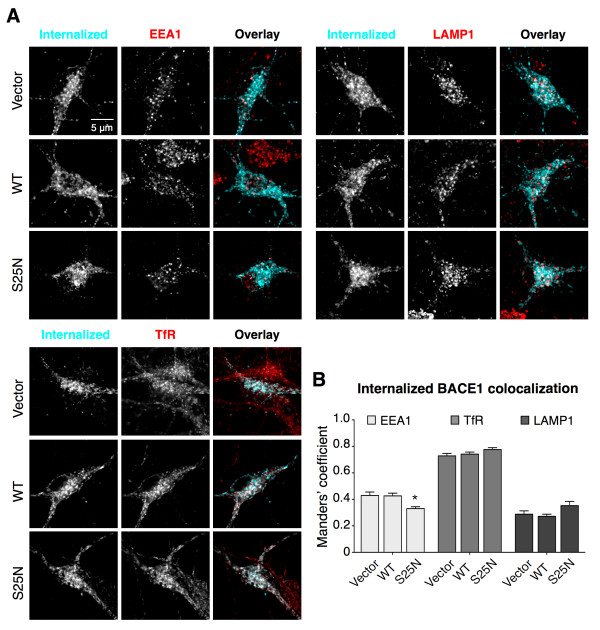
**Confocal analysis of internalized BACE1 localization in the soma of cultured hippocampal neurons. (A)** Representative confocal images of the cell bodies of neurons co-transfected with BBS-BACE1-YFP and the indicated mCherry-tagged Rab11b WT, Rab11_S25N_, or empty vector constructs. Internalized BACE1 pool was labeled by AF647-BTX uptake, and the neurons were fixed following an acid wash to remove remaining surface staining. Neurons were then immunostained with markers of early endosomes (EEA1), lysosomes (LAMP1) and recycling endosomes (TfR), and analyzed by confocal microscopy. **(B)** The extent of colocalization between internalized BACE1 and endogenous organelle markers was quantified in the cell bodies of neurons transfected with the indicated constructs (n = 6-10 neurons each). Note that BACE1 is still able to reach TfR-positive recycling endosomes in the cell body after the overexpression of Rab11_S25N._

In order to determine if dominant-negative Rab11b expression caused an abnormal targeting or retention of BACE1 in cellular organelles in the soma, we performed colocalization analysis of internalized BACE1 with endogenous markers of early endosomes (EEA1), recycling endosomes (transferrin receptor; TfR), and lysosomes (LAMP1), visualized by immunofluorescence staining and confocal microscopy (Figure 7A). We then calculated the Manders’ coefficient to quantify the extent of colocalization. This analysis revealed no evidence of retention of internalized BACE1 in early endosomes and only a small, non-significant increase in localization in lysosomes (Figure 7B). In addition, vesicles containing internalized BACE1 in the soma were largely positive for the recycling endosome marker TfR, regardless of the overexpression of wt Rab11b or Rab11b_S25N_ (Figures 7A and 7B). Thus, we conclude that internalized BACE1 is not grossly mislocalized in either early endosomes or lysosomes upon the expression of Rab11b_S25N_ dominant-negative mutant (Figure 7B). These results indicate that Rab11b GTPase activity does not play a major role in sorting of internalized BACE1 to TfR-positive recycling endosomes in the somato-dendritic compartment. However, axon:dendrite ratio analysis revealed that Rab11 activity is required for efficient axonal targeting of BACE1 (Figure 6D). Together, these results suggest that following internalization BACE1 is targeted to axons in a Rab11-dependent manner.

## Discussion

In this study, we generated transgenic mice expressing BACE1-YFP, and for the first time, visualized BACE1 dynamic axonal transport *in situ* within the mossy fibers of the hippocampus by multiphoton microscopy. Moreover, we investigated the localization and trafficking of BACE1 by live-cell imaging in cultured hippocampal neurons. The dynamic characteristics of BACE1 transport in hippocampal slices and in mature primary cultured hippocampal neurons (DIV12-14) shared several similarities. In both cases we observed two distinct pools of BACE1: a highly dynamic pool of BACE1 found in tubulo-vesicular carriers that were transported bi-directionally and consistent with a microtubule-based transport mechanism; moreover, a second pool of BACE1 found in stationary structures that were relatively larger in size as compared with the motile carriers. FM-dye uptake studies revealed that stationary BACE1-YFP fluorescence corresponds to active presynaptic terminals. Finally, using immunofluorescence labeling and dual-color imaging, we demonstrate BACE1-YFP localization and dynamic transport in Rab11-positive recycling endosomes. Expression of a dominant-negative Rab11 mutant causes accumulation of internalized BACE1 in the soma concomitant with a loss of BACE1 levels in the axons, consistent with BACE1 transcytosis from the somato-dendritic compartments to the axons in endosomes.

In human brain, BACE1 can be observed by immunostaining in dendrites of CA1 neurons [34]. Interestingly, in mouse brain, endogenous BACE1 is highly enriched in hippocampal mossy fiber terminals, and only low levels of BACE1 can be detected in the neuronal soma and dendrites (Figure 1) [33,34]. These apparent differences likely represent the balance between the biosynthetic level and the efficiency of the transport machinery responsible for BACE1 trafficking. Thus, overexpression of BACE1-YFP in cultured hippocampal neurons allows us to appreciate dynamic sorting of BACE1 between dendrites and axons, which is not possible to discern from steady-state analysis of endogenous proteins that have reached their preferred final destination. Indeed, our results on BACE1 localization are analogous to what has been documented for the localization of kainate-type glutamate receptor subunits GluK2 and GluK3. When analyzed by immunohistochemistry, the distribution of GluK2 and GluK3 is prominent at hippocampal mossy fiber synapses in mouse brain [44]. However, detailed analysis of these receptors by transfection in cultured hippocampal neurons reveals a prominent dendritic localization and demonstrates that the steady-state localization of kainate receptors is achieved by dynamic activity-dependent polarized endocytic sorting from the dendrites through recycling or degradative pathways [45,46]. The progressive increase in BACE1 levels in axons concomitant with its decrease in dendrites over time following transfection suggest the existence of saturable mechanisms that mediate polarized axonal sorting of BACE1 (Figure 3). Axonal sorting and presynaptic localization of BACE1 are highly relevant because of the recent identification of several presynaptic proteins such as L1, CHL1, LRRN1, brain EGF-repeat containing transmembrane protein, and neurexin-1a as neuronal BACE1 substrates [47,48]. Moreover, loss of BACE1 expression results in axon guidance defects in the hippocampus and the olfactory bulb [36,44,48].

Although the machinery and the molecular mechanisms governing polarized sorting of proteins in neurons needs to be further explored, the involvement of three pathways for the axonal targeting of transmembrane proteins has been suggested: 1) direct polarized delivery of nascent proteins from the secretory pathway to the dendrites or axons in TGN-derived vesicles, 2) non-polarized delivery to dendrites and axons followed by selective retrieval and retention involving endocytosis and degradation pathways, 3) indirect polarized delivery via endosomes, also called transcytosis, whereby nascent proteins are first delivered to the somato-dendritic cell surface, and then following endocytosis get routed to the axons [22]. The extensive localization of BACE1 in endosomes and the highly polarized axonal localization of BACE1 in hippocampal mossy fibers ([34], Figures 1 and 5) suggest that the direct polarized delivery is unlikely to play a major role in BACE1 axonal targeting. The results detailed in this study suggest that BACE1 sorting to Rab11-positive recycling endosomes plays an important role for further transport to the axons and presynaptic terminals. First, BACE1 is efficiently sorted in endosomes positive for three known markers of recycling endosomes: Rab11, syntaxin 13, and TfR [21,34]. Second, the dynamic characteristics of BACE1 transport in hippocampal neurons, characterized by live-cell imaging, are consistent with protein trafficking in recycling endosomes [37], a conclusion supported by two-color imaging of BACE1 co-transport with Rab11 in dendrites and axons (Figure 5, Additional file 4). Third, the impairment of Rab11 activity by dominant-negative mutant expression caused internalized BACE1 accumulation in the soma with a concomitant decrease in axons. The accumulation of internalized BACE1 in the soma and unperturbed relative subcellular distribution in endosomes and lysosomes (Figure 7) suggest that BACE1-containing vesicles are not rerouted for lysosomal degradation when a block in recycling itinerary is imposed by DN Rab11 expression. Because we have not found convincing evidence for the involvement of degradative pathways to explain the paucity of BACE1 in soma and dendrites, we hypothesize that proper BACE1 sorting to axons and presynaptic terminals requires its transcytosis in a Rab11-dependent manner rather than aspecific sorting followed by selective retention/retrieval mechanisms. Interestingly, BACE1 shows only partial colocalization with Rab11 in axons, when observed by immunofluorescence staining or dual-color live imaging (Figure 5). These findings suggest that in axons a subset of BACE1 is sorted independent of Rab11-positive endosomes or Rab11 detaches from a subset of BACE1-positive vesicles. It will be of interest to identify other trafficking adaptors that regulate dynamic BACE1 transport in axons. In this regard, we recently reported that loss of Eps15-homology-domain containing 1 and 3 protein function in hippocampal neurons compromises dynamic axonal transport and overall BACE1 levels in axons [34].

Thus far, only a few proteins have been characterized to undergo neuronal somaodendritic-to-axonal transcytosis; notable examples are the axonal cell adhesion molecule L1/NgCAM [49], tropomyosin-related kinase Trk receptors [41], and Contactin-associated protein 2, Caspr2 [50]. In each case, it is clear that endosomal sorting is required for axonal targeting, but the machinery and the molecular players involved in achieving polarized sorting are not fully understood. Also, differences could be already found between the steady-state localization of these proteins suggesting the existence of different regulatory mechanisms in the transcytotic pathway. For example, L1/NgCAM axonal surface expression is prominent and highly polarized in transfected cultured neurons [49], which is not the case for BACE1. In addition, L1/NgCAM is sorted mainly in NEEP21-positive vesicles in soma/dendrites before transcytosis, and shows only low levels of colocalization with transferrin-positive recycling endosomes. In contrast, a large fraction of BACE1 is found in recycling endosomes positive for TfR and Rab11, with only a relatively smaller fraction present in EEA1 or NEEP21-positive early endosomes (Figure 5, [34]). Thus, it appears that L1/NgCAM and BACE1 are subject to different the endosomal sorting steps prior to transcytosis. BACE1 sorting in recycling endosomes and dynamics share similarities with Trk receptors. Similar to BACE1 trafficking in hippocampal neurons, TrkA is also dynamically co-transported in axons in Rab11-positive vesicles and its trafficking in sympathetic neurons also requires Rab11-GTPase activity for efficient transcytosis [41]. It has been hypothesized that transcytosis serves as a means to facilitate efficient axonal delivery of receptors in response to signals often involving ligand:receptor interaction in presynaptic terminals as it is shown for TrkA transcytosis mediated by nerve growth factor signaling [41]. Since BACE1 is the first reported enzyme to undergo transcytosis, exploring potential signals that regulate BACE1 transcytosis would be of great interest especially for a better understanding of the mechanism underlying its abnormal accumulation in swollen presynaptic terminals in Alzheimer’s disease. Since synaptic activity regulates BACE1 trafficking and APP processing [19-21] it is possible that synaptic activity impairment observed in Alzheimer’s disease could affect BACE1 transcytosis in a manner that promotes its accumulation in dystrophic neurites, thus contributing to local Aβ production near the presynaptic terminals.

Abnormalities of the endosomal system such as the fusion of early and recycling endosomes have long been implicated in Alzheimer’s disease pathogenesis (reviewed in [11]). Interestingly, Rab11 was recently identified as one of the two major regulators of Aβ production, in an unbiased RNAi screen of human Rab GTPases and Rab GTPase-activating proteins by Rajendran and colleagues [51]. Overexpression of dominant-negative mutants of Rab11a or Rab11b also reduced Aβ and sAPPβ levels significantly. Moreover, siRNA knockdown of Rab11 expression in primary neurons significantly reduce sAPPβ and Aβ levels demonstrating that Rab11 function is crucial for β-cleavage and Aβ generation [51]. Our characterization of Rab11 as a novel regulator of BACE1 axonal sorting in neurons, along with the identification of Rab11 as a modulator of Aβ production, raises the possibility that dysfunction of Rab11 may underlie pathogenesis in a subset of sporadic Alzheimer’s disease cases. Indeed, aberrant Rab11 trafficking has been reported in Huntington's disease and contributes to oxidative stress and neuronal cell death [52]. Future studies aimed at manipulating of polarized neuronal BACE1 trafficking to assess potential modulation of Aβ production *in vivo* would be necessary to evaluate if trafficking modulation of BACE1 could serve as a therapeutic strategy in Alzheimer’s disease to reduce cerebral amyloid burden.

## Methods

### cDNA constructs

The expression plasmids that encode BACE1-YFP and BBS-BACE1-YFP (harboring the 13-amino acid α-Bungaratoxin Binding Site) were generated as described in [34]. A similar strategy was used to generate BACE1-Cerulean construct. To generate mouse Rab11b expression plasmids, a mouse brain PCR product that codes for amino acids 72 to 218 was exchanged for the corresponding region in HA-tagged Rab11a WT and Rab11a_S25N_ constructs [53]. The cDNA inserts were then subcloned in-frame into the pmCherry-C1 vector. All constructs were verified by sequencing. pCS2-Cerulean was generated by subcloning the Cerulean coding sequence downstream of the CMV promoter. The Cerulean-tagged synaptophysin expression construct was provided by Dr. Karen L. O'Malley.

### Generation of BACE1-YFP transgenic mice

BACE1 transgenic mice were generated in which expression of BACE1-YFP is regulated by tetracycline [Tet-off system] [54]. A cDNA that encodes human BACE1 fused with YFP through a 22-residue linker was subcloned into the *Eco*RV site of the *tet*O promoter expression vector pMM400 (gift of M. Mayford, The Scripps Research Institute, La Jolla, CA). The *tet*O promoter BACE1-YFP transgene was excised with *Not*I, purified and microinjected into single mouse embryos (Transgenic and Targeted Mutagenesis Lab, NU). *tet*O promoter-BACE1-YFP transgenic lines were established and bred with CAMKIIα promoter-tTA transgenic mice (line B, gift of M. Mayford). Bigenic *tet*O promoter-BACE1-YFP/CamKIIα promoter tTA offspring were identified by PCR, weaned and fed either normal mouse chow or chow containing 200 mg/kg of doxycyline for 4 weeks. Expression of the transgene varied from ~1.5 to >10-fold over endogenous BACE1 (as assessed by Western blot analysis) in bigenic mice derived from different *tet*O promoter-BACE1-YFP lines, but the distribution and localization of BACE1-YFP was similar. Animals from a high-expressor line (#429) was used in this study due to it producing images with the greatest signal to noise ratio.

### Immunohistochemistry and immunofluorescence staining

Brains were harvested from bigenic BACE1-YFP mice (3-month) and WT littermates were fixed overnight at 4°C in 4% paraformaldehyde and cryoprotected in PBS 30% sucrose/PBS. Immunofluorescence staining was performed on 40 μm coronal sections or 30 μm sagittal sections as described [30,55,56]. The following primary antibodies were used: BACE1 rabbit mAb D10E5 (1:125; Cell Signaling), synaptophysin pAb (1:75; R&D System), polyclonal MAP2 (1:250, SantaCruz), MAP2 mAb (1:500; gift of Dr. Lester Binder), and neurofilament mAb NFT160 (1:3,700; Sigma-Aldrich). Alexa Fluor 488-, 568-, or 647-conjugated secondary antibodies (Molecular Probes) were used for detection. Images were acquired on Zeiss LSM 510 META laser scanning confocal microscope (BACE1-YFP transgenic brain in Figures 1C and D) or Leica SP5 II STED-CW Superresolution laser scanning confocal microscope (Figure 1C), and processed using ImageJ software.

### Live imaging on hippocampal slices

Freshly harvested brains of bigenic mice were placed in cold ACSF aerated with 95% CO_2_/5% O_2_ blood gas mixture and cut into 500 μm horizontal slices. The slices were then maintained at room temperature in a bath perfused with aerated ACSF. For live-cell imaging, each slice was positioned on the microscope stage such that the dentate gyrus was on the left side of the image, perfused with ACSF and maintained at 30°C. Slices were excited at 920 nm with Mai-Tai HP DeepSee-OL laser (Spectra-Physics, laser range 690–1040 nm) using a Olympus FV1000MPE multiphoton confocal system mounted on a BX61WI frame. Images were acquired using 60X (NA 1.1) water immersion objective at the rate of 0.8 frame/sec for 4 min.

### Immunofluorescence staining and live cell imaging of primary hippocampal neurons

Hippocampal neurons were cultured from E17 mouse embryos as previously described [34,57]. Dissociated neurons were cultured on poly-D-lysine-coated glass coverslips suspended over a monolayer of primary astrocytes prepared from P0-P2 mouse cortex. Cultures were maintained in Neurobasal supplemented with B27 serum-free and GlutaMAX-I supplement (Invitrogen). Neurons were transfected with Lipofectamine2000 (Invitrogen) on DIV 5 and fixed at various maturation stages in 4% paraformaldehyde containing 4% sucrose. For live-cell imaging, neurons were transfected on DIV11 and used between DIV12-14. The coverslips were maintained in imaging medium (119 mM NaCl, 2.5 mM KCl, 2 mM CaCl_2_, 2 mM MgCl_2_, 30 mM D-glucose, and 25 mM HEPES; pH 7.4) during image acquisition. Functional synapses were labeled in live DIV13 neurons transfected with BACE1-Cerulean by allowing FM_1-43_ Dye uptake, essentially as described [58].

BTX uptake experiments to label internalized BACE1 were performed essentially as described [34]. Briefly, AF647-conjugated BTX (Invitrogen) was added to the culture medium (6.6 μg/ml final), at 37°C for 3 to 4 h. Coverslips were washed with ice-cold Hanks' balanced salt solution with 10 mM HEPES, pH 7.3, and BTX bound to cell surface BACE1 was removed by incubation in an acidic solution (0.5 M NaCl and 0.2 M acetic acid, pH 2.8) for 2 min before fixation.

Fixed neurons on coverslips were quenched with 50 mM of NH_4_Cl for 10 min, permeabilized for 6 min on ice with 0.2% Triton X-100, and blocked with 3% BSA in PBS. The coverslips were incubated for 1 h at room temperature with the primary antibodies diluted in PBS containing 3% BSA: MAP2 mAb (1:5,000; Sigma), EEA1 (1:200; Millipore), Syntaxin 13 (1:1000; Synaptic Systems); TfR mAb (C2F2, 1:250; Pharmingen), rat anti-LAMP1 (ID4B, 1:150; Developmental Studies Hybridoma Bank). Subsequently, the coverslips were incubated with Alexa 555- or 647-conjugated secondary antibodies (Molecular Probes) for 1 h at room temperature and mounted using Permafluor (Thermo Fisher).

### Image acquisition and analysis

Wide-field epifluorescence images of fixed neurons were acquired as 200 nm *z*-stacks using 20X (NA 0.75) or 60X (NA 1.49) objectives. Confocal images were acquired on a Leica SP5 II STED-CW Superresolution Laser Scanning Confocal microscope using 10X (NA 0.4) and 100X (NA 1.4; zoom 2.5) objectives. Image stacks were deconvolved using Huygens software (Scientific Volume Imaging). Extended Depth of Field plugin of ImageJ was used to generate single plane projections from processed *z*-stacks [59]. Quantitative image analysis was performed using Metamorph (Molecular Devices) and ImageJ [60] softwares. Axonal and dendritic BACE1 fluorescence intensities were quantified on 10X single plane images or 60X *z*-stack projections of neurons using an established method as described previously [34,61]. Briefly, the average fluorescence intensities were measured along 100–200 μm-long 1 pixel-wide line segments traced on 2–3 representative sections of dendrites and axons in each neuron using ImageJ. The mean fluorescence intensity in the soma was quantified by drawing a region around the soma. The average axon:dendrite and cell body:dendrite ratios were calculated for each neuron. Normalized axon:dendrite ratio was then calculated by dividing the raw axon:dendrite ratio of BACE1 by axon:dendrite ratio of Cerulean in the same neuron [36]. Manders’ coefficient of colocalization of BACE1 with organelles markers or mcherry-Rab11b was calculated on thresholded confocal 100X images of dendrites and cell bodies, or 60X deconvolved *z*-stack projections of axons (identified by the exclusion of MAP2 staining) using JACoP ImageJ plugin [62].

### Live-cell imaging

Live-cell images were acquired on a motorized Nikon TE 2000 microscope maintained at 37°C in a custom-designed environment chamber, at the rate of 1 frame/sec, using 60X (NA 1.49) objective and Cascade II:512 CCD camera (Photometrics). Dual-color imaging was performed using the Dual View Imaging System (Optical Insights, LLC). Image stacks were processed using ImageJ. Kymographs were generated in Metamorph, and used to determine the frequency and directionality of particle movement, and to quantify maximum velocities of particles moving at the rate of >0.1 μm/sec.

### Statistical analysis

Each experiment was performed using at least three independent sets of cultures unless otherwise specified. Data are presented as mean ^±^ SEM. Statistical significance was determined by t-tests (two groups) or ANOVA (three or more groups) using GraphPad Prism software and indicated the figures: * *p* < 0.05; ** *p* < 0.01; *** *p* < 0.001; **** *p* < 0.0001; *ns* - non-significant.

## Abbreviations

Aβ: β-amyloid; AD: Alzheimer’s disease; AF647-BTX: Alexa-Fluor 647 labeled α-bungarotoxin; APP: Amyloid precursor protein; BACE1-YFP: Yellow fluorescent protein-tagged BACE1; BACE1: β-site APP cleaving enzyme 1; BBS: α-Bungaratoxin Binding Site; BTX: α-bungarotoxin; DN: Dominant-negative; TfR: Transferrin receptor; WT: Wild-type.

## Competing interests

The authors declare that they have no competing interests.

## Authors’ contributions

VBP, CGF, and GT designed experiments, performed live cell imaging, confocal microscopy, and analyzed the data. VBP, CGF, KSV, and JR generated expression plasmids and immunostaining. SR and RV generated and characterized BACE1-YFP transgenic mice. VBP, SR, and JW performed multiphoton imaging. VBP and GT wrote the manuscript. GT conceived of the study, coordinated data analysis, and prepared the final manuscript. All authors read and approved the final manuscript.

## Supplementary Material

Additional file 1 **Time-lapse Analysis of Axonal Transport of BACE1 in Hippocampal Mossy Fibers.** Acute hippocampal slices of BACE1-YFP bigenic mice were maintained at 30°C by perfusion with ACSF, and imaged on Olympus FV1000 MPE multiphoton confocal microscope under 920 nm excitation. Time-lapse images of the hippocampal mossy fibers were acquired at a rate of 0.8 frames/sec (4 μsec/pixel sampling speed) for 4 min, and displayed at 15 frames/sec. BACE1-YFP containing carriers moving away from the hilus (anterograde) or toward the hilus (retrograde) are shown. Representative still images are shown in Figure 2C. Click here for file

Additional file 2 **Dynamic Axonal Transport of BACE1 in Cultured Hippocampal Neurons.** Hippocampal neurons transfected with BACE1-YFP (DIV12) were monitored under a temperature-controlled environment at 37°C on a Nikon TE 2000 microscope. Images were acquired at a rate of 1 frame/sec for 5 min, with an exposure time of 300 ms, and displayed at 10 frames/sec. *Arrows* indicate BACE1 anterograde (blue) and retrograde transport (red). Representative still image and image montages are shown in Figure 4A and 4C. Click here for file

Additional file 3 **Presynaptic Localization of BACE1.** Hippocampal neurons (DIV14) cotransfected with BACE1-YFP and Synaptophysin-Cerulean were observed by live-cell imaging. Sequential time-lapse images were acquired at a rate of 1 frame/sec for 3 min on the same axon, with an exposure time of 300 ms, and displayed at 10 frames/sec. *Arrowheads* point to stationary structures that contain BACE1 and synaptophysin. The anterograde direction of movement is toward the right. Representative still images are shown in Figure 4D. Click here for file

Additional file 4 **Dynamic Colocalization BACE1with Rab11b in Dendrites and Axons.** Hippocampal neurons (DIV14) cotransfected with BACE1-YFP and mCherry-Rab11b were observed by live-cell imaging. Dual-color time-lapse images of a dendritic branch and an axon were acquired using the Dual View imaging system at a rate of 1 frame/sec for 3 min, with an exposure time of 300 ms, and displayed at 7 frames/sec. In the dendrite (top), bidirectional movement of a carrier containing BACE1 and Rab11b, emerging from a larger stationary structure, which also contains both the proteins, is indicated by a *pink arrowhead*; a rapidly moving vesicle containing both BACE1 and Rab11 is indicated by a *blue arrowhead*. In the axon (bottom), *blue and pink arrowheads* indicate rapidly moving carriers that contain both the proteins. The anterograde direction of movement is toward the right. Scale bar 20 μm. Representative kymographs are shown in Figure 5C. Click here for file
